# Prescriptive factors for intensive home treatment in acute psychiatry: a secondary analysis of a randomised controlled trial

**DOI:** 10.1186/s13033-023-00619-1

**Published:** 2024-01-03

**Authors:** Ansam Barakat, Matthijs Blankers, Jurgen E Cornelis, Nick M Lommerse, Aartjan TF Beekman, Jack JM Dekker

**Affiliations:** 1 Department of Research, Arkin Institute for Mental Health Care, Amsterdam, The Netherlands; 2 Department of Psychiatry Amsterdam UMC/VUmc, Amsterdam Public Health research institute Amsterdam UMC, Amsterdam, The Netherlands; 3 Trimbos-Institute, Netherlands Institute of Mental Health and Addiction, Utrecht, The Netherlands; 4 Department of Psychiatry Amsterdam UMC/AMC, Amsterdam Public Health research institute Amsterdam UMC, Amsterdam, The Netherlands; 5 Department of Emergency Psychiatry, Arkin Institute for Mental Health Care, Amsterdam, The Netherlands; 6 Department of Research and Innovation, GGZ InGeest Specialized Mental Health Care, Amsterdam, The Netherlands; 7 Department of Clinical Psychology, Vrije Universiteit Amsterdam, Amsterdam Public Health research institute Amsterdam UMC, Amsterdam, The Netherlands

**Keywords:** Intensive home treatment, Hospitalization, Emergency psychiatry, Moderator analysis

## Abstract

**Background:**

Intensive home treatment (IHT) aims to prevent psychiatric hospitalisation. Although this intervention is well tested, it is still unknown for whom this intervention works best. Therefore, this study aims to explore prescriptive factors that moderate the effect of IHT compared to care as usual (CAU) on symptom severity.

**Methods:**

Using data from a randomised controlled trial, 198 participants that experience an exacerbation of acute psychiatric symptoms were included in this secondary analysis. In order to maximise clinical relevance, generally available environmental and clinical baseline factors were included as tentative moderators: age, gender, employment status, domestic situation, psychiatric disorders, psychological symptoms, psychosocial functioning, alcohol and other substance use. The outcome variable symptom severity was measured using the Brief Psychiatric Rating Scale (BPRS) and collected at 26 and 52 weeks post-randomisation. Multiple regression analysis was used to examine which participants’ characteristics moderate the effect of IHT on the total BPRS score.

**Results:**

Our results suggest that being employed (B = 0.28, SE = 0.13, 95% CI = 0.03–0.53, *p* = 0.03) at baseline seems to have a moderation effect, which result in lower symptom severity scores at 26 weeks follow-up for patients who received IHT. This effect was not found at 52 weeks.

**Conclusions:**

On the basis of the number of factors tested, there is no evidence for robust outcome moderators of the effect of IHT versus CAU. Our conclusion is therefore that IHT can be offered to a diverse target population with comparable clinical results.

**Trial registration:**

This trial is registered (date of registration: 2016-11-23) at the international clinical trials registry platform (NTR6151).

**Supplementary Information:**

The online version contains supplementary material available at 10.1186/s13033-023-00619-1.

## Background

In recent years, several initiatives have been developed to reduce or prevent hospital admission for patients in acute psychiatry. Although hospitalisation may be necessary and helpful in many cases [1, 2], many studies have shown that it can be harmful and stigmatizing to patients as well [3–5]. Alternatives to inpatient care are available [6, 7] such as treatment by outpatient Crisis Resolution Teams (CRTs) [8–11] which are named Intensive Home Treatment (IHT) teams in the Netherlands [12]. In a recent study, IHT was found to be efficacious in reducing hospital admission and inpatient bed usage [13]. Our study showed that after 12 months, the mean number of admission days in the intensive home treatment condition was 42.47 (SD = 53.92) versus 67.02 (SD = 79.03) for care as usual, a reduction of 36.6% (*p* = 0.03). These findings are in line with two previous randomised controlled studies on similar interventions [14, 15]. Regarding clinical recovery, we have not found significant differences between IHT and care as usual (severity of mental health symptoms was measured using the Brief Psychiatric Rating Scale after 12 months; the IHT condition mean item score 1.65 (SD = 0.55) versus 1.65 (SD = 0.50) for care as usual, *p* = 0.97) [13]. These findings were supported by other studies who found that patients who received home treatment had personalised care and were found to have a clinical recovery similar to hospital admission [14, 16, 17]. In recent years, and supported by the accumulating evidence base, the use of IHT is spreading [16, 18, 19].

IHT is part of the acute psychiatry services and is offered to patients that experience a psychiatric crisis. The patients presented at acute psychiatry services vary with regard to both their clinical symptoms and their contextual circumstances. It is unlikely that IHT is a magic bullet that has similar benefits for all. Therefore, it is important to explore whether specific subgroups of patients might benefit more (or less) from IHT in comparison to other forms of crisis care. Research about different types of subgroups can provide important information for future clinical trials and for routine clinical practice [20, 21]. Nevertheless, there is a lack of studies exploring the effect of such subgroups on IHT. To identify for whom an intervention works best, potential prescriptive factors should be evaluated. In this secondary analysis of our trial data, we aim to test a number of putative clinical and environmental moderators of the effects of IHT compared to care as usual (CAU) in acute psychiatry, with severity of psychiatric symptoms as the outcome.

## Methods

### Design

This study draws from data collected in the context of a randomised controlled trial (RCT) in which 246 patients participated to compare the effects of IHT and CAU. The trial was registered in the international clinical trials registry platform (ID NTR6151) and the Dutch Union of Medical-Ethic Trial Committees approved the study design (The Medical Ethics Committee of VU University Amsterdam #NL55432.029.16). The design, rationale and outcome of the RCT has been presented elsewhere [22]. This study has been performed in accordance with the Helsinki Declaration as revised in 1989.

### Patients and procedure

Patients were enrolled from two mental health organisations that provide high-intensity psychiatric care in the city of Amsterdam. Patients were recruited by IHT teams and from psychiatric wards between November 2016 and November 2018. Patients included in the RCT were 18 to 65 years of age, experiencing an acute psychiatric crisis severe enough to warrant hospital admission as indicated by a psychiatrist, having been diagnosed with at least one DSM-IV-TR or DSM-5 disorder (not primarily a substance use disorder), and residents of Amsterdam, the Netherlands. Patients were excluded if they lacked basic knowledge of the Dutch language, were homeless, or had previously received IHT. Moreover, patients who receive (Flexible) Assertive Community Treatment care ([FACT) were excluded as they already receive ongoing care [23]. These (F)ACT teams can up-scale their care by providing more frequent house visits when the patient’s condition deteriorates.

During the first contact with professionals, patients who met the study criteria were pre-randomised to IHT or CAU following a Zelen double consent open-label design [24]. For the allocation of the patients, seeded pseudo-random number generator was used for randomisation. The seed was based on patient characteristics so that if the screen and randomisation tool was incidentally completed more than once for the same patient, the same allocation would result. The applied allocation ratio was 2:1 for reasons of staff and facility capacity. Before participation, an independent psychiatrist assessed patients’ mental capacity to provide consent for research. Patients not considered mentally competent were not included in the study. According to the Zelen double consent design, all participants were fully informed about the study during the first meeting with a member of the research group and before consent is sought. This implies that patient got information about treatment allocation based on pre-randomisation, study procedures, treatment options, and the possibility to cross-over to the other treatment condition, before they were asked to sign the informed consent form. Only patients who provide written in- formed consent were included in the study. Patients could partake in interviews, share their medical records, or both. The interviews were conducted at baseline, 6 weeks post-baseline, and 26, and 52 weeks post-randomisation. For this study, the baseline, 26 and 52 weeks follow-up measurements were used.

### Interventions

IHT is an intensive short-term outpatient treatment intervention which provides intensive care more than twice a week and continues for an average of six weeks until a crisis is resolved. IHT teams are multidisciplinary and act as gatekeepers for psychiatric hospitalisation by assessing every patient while balancing the necessity of hospitalisation and the possibilities of IHT. For patients who have initially been hospitalised, IHT starts as soon as discharge is considered.

CAU consisted of all commonly available psychiatric crisis resolution treatments except IHT. This includes allocation to specialised mental health care hospital or other less intensive outpatient care (i.e. two times a week or less). The treatment groups have been described in more detail elsewhere [22].

### Treatment outcome measure

The severity of mental health symptoms was measured using the Brief Psychiatric Rating Scale (BPRS) [25]. The BPRS is an interview-based questionnaire that consists of 24-items; each item can be scored on a seven point scale ranging from 1, ‘not present ’ to 7, ‘extremely severe’ and has a recall period of four weeks. This instrument has an internal consistency of α = 0.68–0.74 [25]. Higher scores on the BPRS are indicative of higher severity of mental illness.

### Prescriptive factors evaluated as moderators

The choice of the prescriptive factors was made before the study was conducted, based on previous studies [26–31] and prescriptive factors being available in routine clinical care data. All included prescriptive factors were measured at baseline.

Demographic and environmental prescriptive factors include age, gender (0 = female, 1 = male), employment status (0 = unemployed, 1 = employed), and domestic situation (0 = living alone, 1 = living with others). The type of psychiatric disorders were extracted from the electronic patient record system. Psychological symptoms severity was evaluated using the Brief Symptom Inventory (BSI) questionnaire [32]. This questionnaire assesses clinically relevant symptoms in the week prior to data collection and consists of a 53-item. This instrument has a range score of 0 to 4. Higher scores on the BSI indicate more severe symptoms. The Health of the Nation Outcome Scales (HoNOS) was used to assess behaviours, impairment, symptoms and social functioning in the two weeks prior to the interview [33]. The HoNOS is a clinician rated instrument and was collected from the electronic patient record system. This instrument consists of a 12-item, total HoNOS score has a range score of 0 to 48. Higher scores on the HoNOS indicate poorer psychosocial functioning. Assessments of aggression (item 1) and suicidality (item 2) were individual HoNOS items, these items had a range score of 0 to 4. Alcohol use in the previous twelve months was measured using the Alcohol Use Disorder Identification Test (AUDIT) [34]. This instrument screens for problematic alcohol use, defined as risky or hazardous consumption or (any) alcohol dependency. Substance use in the previous thirty days was assessed with the Measurements in the Addictions for Triage and Evaluation (MATE) Module 1 [35]. This interview based questionnaire assesses the person’s use of psychoactive substances both in the past 30 days and average usage on a typical day.

### Statistical analysis

All patients included in the RCT were analysed according to the originally allocated treatment group. The RCT was powered to test the effect of IHT on the number of inpatient days (primary outcome) [13] and not specifically to be able to test for effect modification.

Patients’ characteristics were analysed using a standard approach including descriptive statistics and appropriate statistical tests. Multiple regression analysis was used to examine which patients’ characteristics moderate the effect of the intervention on symptom severity. Assumptions for regression analyses (linearity, homoscedasticity, normality of the residuals, absence of multicollinearity) were checked and met. For each potential moderator variable a separate regression model was fitted. Each model included baseline BPRS symptom severity, a treatment condition dummy variable (0 = CAU; 1 = IHT), the baseline value of the evaluated prescriptive variable and the prescriptive variable-by-treatment condition interaction term. Pairwise deletion was used for missing data.

All statistical analyses were performed using SPSS Statistics software version 27.0 for Windows (IBM Corp. Released 2020) and all two-sided statistical tests were performed with a significance level of α < 0.05.

## Results

### Characteristics of the patients

In total, 246 patients participated in the RCT of whom 48 gave permission to use their medical records but did not participate in the interviews. As no BPRS data was available for those 48 patients, a total of 198 patients (IHT n = 146, CAU n = 52) could be included in the current study (see additional file 1). Pearson’s chi-square and t-tests indicated no differences between the interviewed sample (n = 198) and the medical records only sample (n = 48) regarding age, gender, country of birth, education, domestic situation, employment status and the symptom severity and social functioning administered by the Health of the Nation Outcome Scales (HoNOS) at baseline (all *p* ≥ 0.05).

Patients’ *sociodemographic* characteristics at baseline are presented in Table 1. Patients included in this study were between 18 and 65 years old (mean = 40.65, SD = 12.48), 108 (54.5%) were female. Patients’ clinical characteristics at baseline are presented in Table 2. The most frequent diagnoses were depressive disorders (22.7%), dipolar disorders (21.7%) and schizophrenia spectrum and other psychotic disorders (34.3%). Other disorders included mood disorder not otherwise specified (nos) (n = 1), autisme spectrem disorder (n = 3), RETT syndrome (n = 1), panic disorder nos (n = 3) and without agorofobia (n = 3), obsessive compulsive disorder (n = 1), acute stress disorder (n = 1), trauma and stressor related disorder (n = 4) and deficit hyperactivity disorder (n = 1). Baseline total score of the BPRS was on average 1.78 (SD = 0.40). No significant differences were found between patients who received IHT and CAU for both clinical and environmental prescriptive factors (all *p* > 0.05), except for domestic situation (*p* = 0.01). The CAU group consisted of patients who mainly lived alone (57.7%); patients in the IHT group often lived with others (62.5%).

**Table 1:** Participants’ sociodemographic characteristics at baseline

	All participants	Intensive home treatment	Care as usual	*p* value*
Age, mean (SD)	40.65 (12.48)	39.72 (12.56)	43.27 (11.99)	0.08
Gender, n (%)				0.33
Female	108 (54.5)	83 (56.8)	25 (48.1)	
Male	90 (45.5)	63 (43.2)	27 (51.9)	
Domestic situation, n (%) ^a^				0.01
Living alone	84 (42.9)	54 (37.5)	30 (57.7)	
Living with others	112 (57.1)	90 (62.5)	22 (42.3)	
Employed, n (%) ^*b*^	99 (50.3)	75 (51.7)	24 (46.2)	0.52

* Fishers exact Test was used for categorical variables. ^*a*^*n = 144 (IHT);*^*b*^*n = 145 (IHT).*

**Table 2:** Participants’ clinical characteristics at baseline

	All participants	Intensive home treatment	Care as usual	*p* value*
Mental disorders, n (%)				0.99
Depressive disorders	45 (22.7)	33 (22.6)	12 (23.1)	
Bipolar disorders	43 (21.7)	31 (21.2)	12 (23.1)	
Schizophrenia spectrum and other psychotic disorders	68 (34.3)	52 (35.6)	16 (30.8)	
Personality disorders	13 (6.6)	9 (6.2)	4 (7.7)	
Substance use disorders	8 (4.0)	6 (4.1)	2 (3.8)	
Other disorders	18 (9.1)	13 (8.9)	5 (9.6)	
No diagnosis	3 (1.5)	2 (1.4)	1 (1.9)	
Psychological symptoms (BSI total score), mean (SD) ^*a*^	1.06 (0.77)	1.11 (0.76)	0.94 (0.78)	0.22
Psychosocial functioning (HoNOS total score), mean (SD) ^b^	14.27 (6.02)	14.36 (6.28)	13.87 (4.96)	0.78
Suicidality (HoNOS item 2), mean (SD) ^b^	0.77 (1.19)	0.78 (1.19)	0.73 (1.22)	0.89
Aggression (HoNOS item 1), mean (SD) ^b^	1.49 (1.18)	1.45 (1.19)	1.67 (1.11)	0.53
Alcohol consumption (AUDIT), mean (SD) ^c^	5.49 (6.10)	5.48 (6.10)	5.54 (6.18)	0.95
Substance use (MATE), n (%) ^*d*^				
Cannabis	51 (25.8)	39 (27.5)	12 (23.1)	0.59
Other substances	15 (7.6)	12 (8.2)	3 (5.8)	0.76
Symptom severity (total BPRS), mean (SD) ^*e*^	1.78 (0.40)	1.81 (0.41)	1.69 (0.35)	0.07

* Fishers exact Test was used for categorical variables ^*a*^*n = 131 (IHT) 45 (CAU);*^*b*^*n = 64 (IHT) 15 (CAU);*^*c*^*n = 138 (IHT) 50 (CAU);*^*d*^*n = 142 (IHT) 52 (CAU);*^*e*^*n = 137 (IHT) 48 (CAU).*

### Effect of prescriptive factors

Table 3 summarises the associations found between prescriptive factors and symptom severity (measured by total BPRS) at 26 and 52 weeks after randomisation. The percentage of the patients who completed the interviews at 26 and 52 weeks post-randomisation was 82.3% and 76.8%, respectively. The interaction effect between employment status and the treatment group was statistically significant at 26 weeks (B = 0.28, SE = 0.13, 95% CI = 0.03–0.53, *p* = 0.03), but not at 52 weeks (*p* = 0.36). In addition, we found no significant moderator effect for age, gender, domestic situation or type of diagnosis at either 26 and 52 weeks. None of the models involving clinical prescriptive factors indicated a significant moderation effect.

**Table 3:** The relation between prescriptive factors and the effect of IHT on the severity of psychiatric symptoms at 26 and 52 weeks

	26 weeks					52 weeks				
	n	B	SE	95% CI	*p* value	*n*	B	SE	95% CI	*p* value
**Age**	154	< 0.01	< 0.01	< 0.01–0.01	0.05	145	< 0.01	<-0.01	< 0.01–0.01	0.15
Interaction term		-0.01	0.01	-0.02–0.01	0.12		-0.01	0.01	-0.02–0.01	0.30
**Gender** (female)	154	0.07	0.07	-0.06 – 0.21	0.28	145	0.01	0.07	-0.12–0.14	0.89
Interaction term		-0.08	0.13	-0.33–0.18	0.54		0.09	0.12	-0.15–0.34	0.45
**Domestic situation** (living alone)	154	0.02	0.07	-0.12–0.15	0.79	145	0.08	0.07	-0.05–0.21	0.21
Interaction term		0.09	0.13	-0.17–0.35	0.50		-0.20	0.13	-0.44–0.05	0.12
**Employment** (unemployed)	154	0.04	0.07	-0.08–0.17	0.50	145	0.01	0.06	-0.12–0.13	0.93
Interaction term		0.28	0.13	0.03–0.53	0.03		0.11	0.12	-0.13–0.36	0.36
**Mental disorders**	154					145				
**Depressive disorders**		-0.06	0.11	-0.29–0.16	0.57		-0.02	0.10	-0.22–0.18	0.85
Interaction term		< 0.01	0.21	-0.42–0.41	0.99		-0.02	0.19	-0.40–0.36	0.91
**Bipolar disorders**		-0.14	0.11	-0.37–0.09	0.22		-0.15	0.10	-0.35–0.06	0.16
Interaction term		-0.01	0.21	-0.42–0.40	0.96		0.13	0.19	-0.25–0.51	0.51
**Schizophrenia spectrum and other psychotic disorders**		-0.21	0.10	-0.41 – -0.01	0.04		-0.18	0.09	-0.37–0.01	0.06
Interaction term		0.36	0.19	-0.02–0.74	0.07		0.19	0.18	-0.18–0.55	0.32
**Personality disorders**		-0.04	0.16	-0.36–0.27	0.79		0.14	0.15	-0.16–0.44	0.37
Interaction term		0.01	0.29	-0.56–0.58	0.98		-0.07	0.27	-0.61–0.47	0.80
**BSI total score**	148	0.12	0.05	0.03–0.22	0.01	138	0.15	0.05	0.06–0.24	< 0.01
Interaction term		-0.02	0.08	-0.19–0.14	0.78		0.04	0.08	-0.12–0.20	0.61
**HoNOS total score**	67	0.01	0.01	-0.01–0.03	0.27	60	0.01	0.01	-0.01–0.02	0.25
Interaction term		-0.04	0.02	-0.09 – < 0.01	0.06		0.01	0.02	-0.03–0.05	0.70
**Aggression** (HoNOS 1)	67	-0.01	0.04	-0.09–0.07	0.77	60	-0.02	0.04	-0.10–0.06	0.61
Interaction term		-0.10	0.11	-0.32–0.12	0.37		-0.05	0.10	-0.24–0.15	0.63
**Suicidality** (HoNOS 2)	67	-0.02	0.04	-0.10–0.07	0.68	60	< 0.01	0.04	-0.07–0.07	1.00
Interaction term		-0.09	0.09	-0.27–0.10	0.35		-0.04	0.08	-0.19–0.12	0.64
**AUDIT total score**	148	-0.01	0.01	-0.02 – < 0.01	0.12	139	< -0.01	0.01	-0.01–0.01	0.67
Interaction term		< 0.01	0.01	-0.02–0.03	0.81		0.02	0.01	< 0.01 − 0.04	0.05
**Cannabis** (no cannabis used)	152	0.11	0.08	-0.04–0.26	0.15	143	0.01	0.08	-0.14–0.16	0.93
Interaction term		-0.25	0.15	-0.54–0.04	0.10		-0.01	0.15	-0.30–0.28	0.94
**Other substances** (no drugs used)	154	0.23	0.13	-0.03–0.49	0.08	145	0.19	0.12	-0.05–0.43	0.13
Interaction term		-0.36	0.25	-0.85–0.12	0.14		-0.42	0.23	-0.87–0.04	0.07

All models included the treatment variable (CAU/IHT) and baseline total BPRS. IHT group was the reference variable for the intervention. The category other disorders was used as the reference variable. CI = Confidence interval. Interaction term = prescriptive variable-by-treatment condition.

## Discussion

A number of clinical and environmental variables were tested for moderating the effect of IHT versus CAU. Among the tested variables, only employment status was found to moderate between IHT and CAU effectiveness at the 26 weeks follow-up. No significant prescriptive factors were found at 52 weeks. Given the number of tests conducted [36], a fair conclusion is that no firm evidence for prescriptive factors was found.

Despite that we found no prescriptive factor that reliably moderated the effect of IHT on symptom severity, it is worthy to point out that being unemployed (*p* = 0.03) at baseline seems to have a moderation effect, which result in higher symptom severity scores at 26 weeks follow-up for patients who received CAU. We have not found any previous studies who look at prescriptive factor that moderated the effect of IHT on symptom severity. Yet, previous prediction studies have identified unemployed patients to have a higher risk for being admitted to a psychiatric hospital [26, 29, 31]. Employment of the patient might offer IHT-teams the possibility to offer more structure in the patients’ daily routines, and thus might be an indicator for selection to offer IHT. Yet this hypotheses needs to be tested first.

We also found that patients with a diagnosis of schizophrenia or a related disorder (*p* = 0.07) at baseline seems to have a moderation effect, which result in higher symptom severity scores at 26 weeks follow-up for patients who received CAU. In a recent cohort study, IHT was found to be effective in reducing the severity of illness and improving the clinical condition for patients with acute schizophrenia [37]. Furthermore, we found a statistical trend in case of poorer psychosocial functioning (total HoNOS score, 26 weeks *p* = 0.06 ) towards less severe symptoms for patients who received CAU as opposed to IHT. The same account for patients who used drugs or had more problems with alcohol use before baseline (52 weeks *p* = 0.07), we found a statistical trend towards less severe symptoms when those patients received CAU. Due to the dearth of research on the potential effects of the aforementioned prescriptive factors as possible moderators of IHT, additional research is needed to conform and further evaluate our findings.

### Strengths and limitations

A strength of our study was the fact that we were able to use recent data from a pragmatic RCT with high follow-up response rates that was carried out in the acute psychiatry setting in Amsterdam. Our study was primarily powered to test effects of IHT on the number of hospitalisation days. Consequently, statistical power for the moderator analyses was somewhat limited. Secondly, the effect of psychosocial functioning as prescriptive factors for IHT was based on a small proportion (n = 67) of patients. To confirm the lack of moderating effect of psychosocial functioning or other prescriptive factors future studies could include more patients to enable them to perform a moderator analysis and additional subgroup analyses with more statistical power [36]. However, including more patients from this target population is difficult [13]. More research about different prescriptive factors should be conducted in various studies. Those various studies could together contribute to more knowledge.

## Conclusions

In conclusion, we found no convincing evidence that clinical or environmental prescriptive factors moderate the effect of IHT versus CAU on the severity of psychiatric symptoms. It appears that IHT can be offered to a diverse target population with relatively comparable clinical results.

### Electronic supplementary material

Below is the link to the electronic supplementary material.

Supplementary Material 1

## Data Availability

The data that support the findings of this study are available from the corresponding author, AB, upon reasonable request.

## List of abbreviations

CRTs: Crisis Resolution Teams
CAU: care as usual
IHT: Intensive Home Treatment
RCT: randomised controlled trial
(F)ACT: (Flexible) Assertive Community Treatment care
BPRS: Brief Psychiatric Rating Scale
BSI: Brief Symptom Inventory
HoNOS: The Health of the Nation Outcome Scales
AUDIT: Alcohol Use Disorder Identification Test
MATE: the Measurements in the Addictions for Triage and Evaluation
