# Supplementary figures and images for: Prescriptive factors for intensive home treatment in acute psychiatry: a secondary analysis of a randomised controlled trial
Source: Int J Ment Health Syst. 2024 Jan 3;18:2. doi: 10.1186/s13033-023-00619-1 (PMC10763431; doi:10.1186/s13033-023-00619-1)

**Figure 1: Flowchart pre-randomisation and inclusion.**

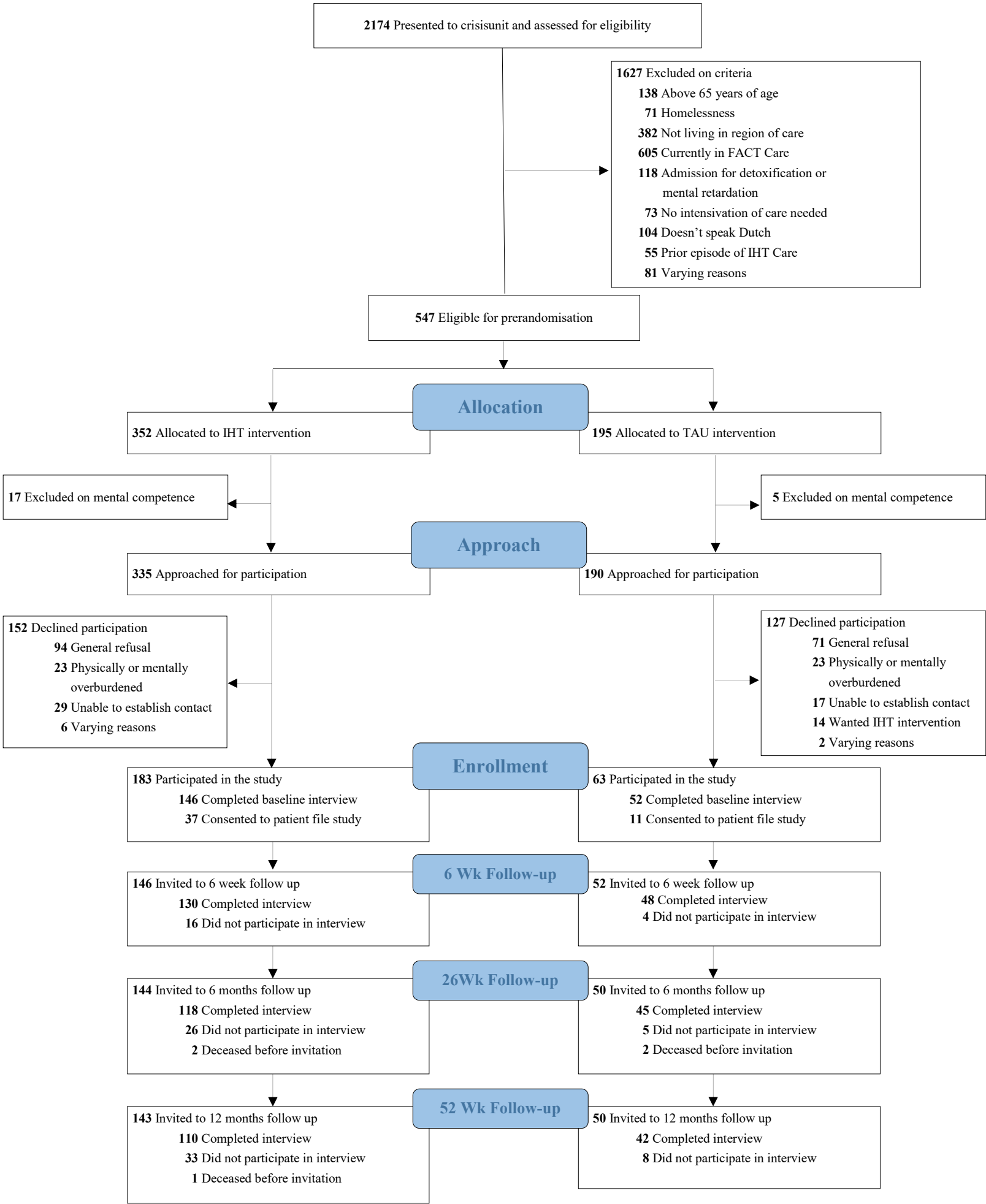

Supplement: Supplementary file 1 — Supplementary Material 1 [file 13033_2023_619_MOESM1_ESM.pdf]
